# Research of recombinant influenza A virus as a vector for *Mycoplasma pneumoniae* P1a and P30a

**DOI:** 10.1002/iid3.70021

**Published:** 2024-09-18

**Authors:** Liang Yu, Wang Yongbo, Yang Shengjun, Tan Jia, Xu Ya, Liao Guoyang, Ma Linna

**Affiliations:** ^1^ Department of Clinical Laboratory The First People's Hospital of Yunnan Province Kunming China; ^2^ The Fifth Department of Biological Products Institute of Medical Biology, Chinese Academy of Medical Science and Peking Union Medical College Kunming China; ^3^ Department of Medical Laboratory Technique Kunming Medical University Haiyuan College Kunming China

**Keywords:** genetic engineering, influenza virus vector, Mycoplasma pneumoniae, vaccine

## Abstract

**Background:**

*Mycoplasma pneumoniae* (*MP*) is a common respiratory pathogen affecting the longevity of the elderly and the health of children. However, the human vaccine against MP has not been successfully developed till now due to the poor immunogenicity and side effects of *MP* inactivated or attenuated vaccine. Therefore, it is necessary to develop a *MP* genetic engineering vaccine with influenza virus strain as vector.

**Methods:**

In this study, the major antigen genes P1a of *MP* adhesion factor P1(3862‐4554 bases) and P30a of P30(49‐822 bases) were inserted into the nonstructural protein (NS) gene of Influenza A virus strain A/Puerto Rio/8/34(H1N1), PR8 for short, to construct the recombinant vectors NS‐P1a or NS‐P30a. The recombinant pHW2000 plasmids containing NS‐P1a or NS‐P30a were cotransfected with the rest 7 fragments of PR8 into HEK293T cells. After inoculating chicken embryos, the recombinant influenza viruses rFLU‐P1a and rFLU‐P30a were rescued. RT‐PCR and sequencing were used to identify the recombinant viruses. The hemagglutination titers of rFLU‐P1a and rFLU‐P30a were determined after five successive generations in chicken embryos so as to indicate the genetic stability of the recombinant viruses. The morphology of recombinant influenza viruses was observed under electron microscopy.

**Results:**

P1a or P30a was designed to be inserted into the modified NS gene sequence separately and synthesized successfully. RT‐PCR identification of the recombinant viruses rFLU‐P1a and rFLU‐P30a showed that P1a (693 bp), P30a (774 bp), NS‐P1a (1992bp) and NS‐P30a (2073 bp) bands were found, and the sequencing results were correct. After five successive generations, each virus generation has a certain hemagglutination titer (from 1:32 to 1:64), and the band of P1a or P30a can be seen in the corresponding positions. The virus particles under the electron microscope appeared as spheres or long strips connected by several particles, revealing a complete viral membrane structure composed of virus lipid bilayer, hemagglutinin, neuraminidase, and matrix proteins.

**Conclusion:**

The recombinant viruses rFLU‐P1a and rFLU‐P30a which carried the advantaged immune regions of the P1 and P30 genes in MP were successfully constructed and identified. And the genetic stability of rFLU‐P1a or rFLU‐P30a was relatively high. The typical and complete morphology of influenza virus was observed under the electron microscope. Our research provided a foundation for the further development of *MP* vaccines for human.

## Graphical abstract

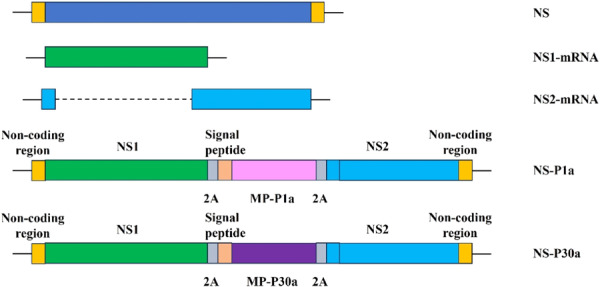

## 1 INTRODUCTION

Mycoplasma is the smallest prokaryotic cell microorganism that lacks a cell wall and can be cultured and proliferated in artificial medium.^1^ The pathogenic mycoplasma to humans mainly include *Mycoplasma pneumoniae* (MP), Ureaplasma urealyticum, *Mycoplasma hominis*, and *Mycoplasma genitalium*.^2^ MP is one of the most important pathogens that can lead to both acute upper and lower respiratory tract inflammation as well as extrapulmonary syndromes, which affecting the longevity of the elderly and the health of children especially.^3^, ^4^ MP can directly harm the respiratory epithelium via adhesion and cytotoxicity, and can also cause pneumonia and other systemic damage through immune dysfunction.^5^

Owing to the absence of a cell wall in MP, the primary therapeutic agents for MP are macrolides that suppress protein synthesis. With the widespread application of antibiotics, there has been an emergence of macrolide‐resistant Mycoplasma pneumoniae (MRMP), presenting significant challenges for the control and treatment of MP infections.^6^, ^7^ Therefore, the emergence of MRMP further emphasizes the role of vaccines as a crucial strategy for preventing and controlling MP infection.^8^ However, the human vaccine against MP has not been successfully developed in the world. The reasons include the poor immunogenicity and poor protective effect of MP inactivated vaccine and subunit vaccine, and side effects of residual virulence and reversion of virulence of MP attenuated vaccine.^9^ Meanwhile, no MP vaccines are currently available partly due to Mp Vaccine‐Enhanced Disease (VED). Research showed that 36% of inactivated MP‐vaccinated individuals exhibited more severe clinical symptoms upon challenge than those receiving a placebo.^10^ Another reseach showed that vaccination of BALB/c mice with MP lipid‐associated membrane proteins (LAMPs) resulted in lipoprotein‐dependent VED after challenge with virulent MP.^11^ Therefore, this study proposed a new idea to develop MP genetic engineering vaccine with influenza virus strain as a vector. Influenza A virus strain A/Puerto Rio/8/34(H1N1), PR8 for short, is a chicken embryo adapted strain, which is recommended by the World Health Organization (WHO) as the donor strain of influenza virus vaccine strain.

The adhesion organelles of MP are composed of adhesion proteins, accessory proteins, and molecular chaperones. Its adhesion to lung ciliated epithelial cells is the first step and one of the main pathogenic mechanisms of MP infection. P1 and P30 are the principal adhesion proteins in the MP infection process, and their respective coding genes are situated in regions of high conservation.^12^ In this study, the immune dominant fragments (P1a, P30a) of MP P1 and P30 were inserted into the NS1 gene to construct recombinant NS gene by utilizing the ability of NS1 gene to accommodate exogenous genes and the self‐lytic property of hand‐foot‐mouth virus 2 A protein.^13^ The reverse genetic manipulation technique of PR8 influenza virus 8 plasmid (pHW2000) system was used to rescue recombinant influenza virus, which contained P1 and P30 immunodominant fragments P1a and P30a. Similar to the intranasal influenza vaccines, it has the potential being used intranasally to specifically induce mucosal immunity in the respiratory tract, and has great value in the development and production of MP vaccine in future.^14^

## 2 METHODS

### 2.1 Cells and viruses

The influenza virus H1N1 (A/Puerto Rico/8/34) (hereinafter referred to as PR8), preserved and used by the Liao Guoyang research group of the Institute of Medical Biology, Peking Union Medical College, Chinese Academy of Medical Sciences. It can provide a total of 8 gene fragments, including PB2, PB1, PA, HA, NP, NA, M, and NS. Human embryonic kidney cells(HEK293T) were purchased from Beina Biology Co., Ltd. (number: BNCC100530) and maintained in Dulbecco's modified Eagle's medium (DMEM) (Sigma Aldrich) supplemented with 10% fetal bovine serum (FBS) (Gibco) and penicillin–streptomycin.

### 2.2 Construction of plasmids

**Figure 1:**
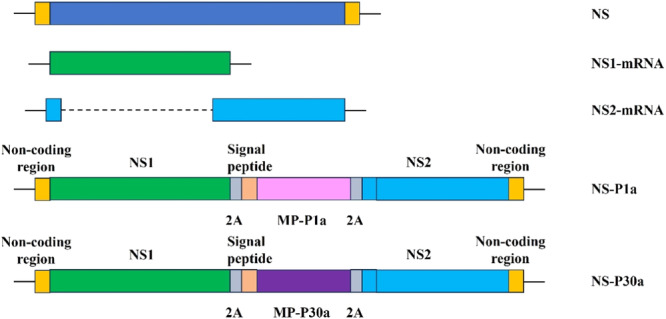
Construction of NS‐P1a or NS‐P30a.

**Figure 2:**
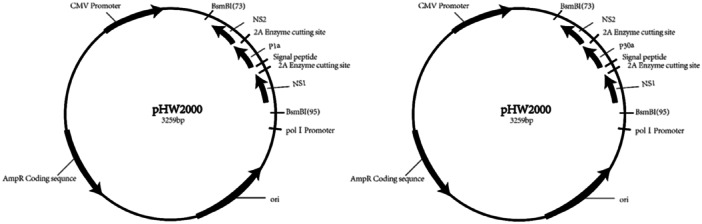
Schematic diagram of pHW‐NS‐P1a and pHW‐NS‐P30a.

P1a is the advantaged immune region (3862‐4554 bases) of the P1 gene in MP (ATCC29342/M129 strain), and P30a is the advantaged immune region (49–822 bases) of the P30 gene in MP.^15^ P1a or P30a was designed to be inserted into the modified NS gene sequence separately and synthesized by Shanghai Shenggong Biotechnology Co., Ltd. The modified NS genes mentioned above include the noncoding region of NS, NS1 gene, 2 A restriction site of Hand, Foot and Mouth Disease (HFMD) virus, signal peptide, P1a/P30a, NS2 gene, etc., as shown in the following figure (Figure 1). We obtained pHW2000 vectors containing various gene fragments of H1N1 PR8 through homologous recombination method with ClonExpress® II One Step Cloning Kit (Vazyme Biotech),^16^, ^17^ including pHW‐PB2, pHW‐PB1, pHW‐PA, pHW‐HA, pHW‐NP, pHW‐NA, pHW‐M, pHW‐NS‐P1a or pHW‐NS‐P30a (Figure 2). After ligation, they were transformed into DH5α competent cells and recombinant plasmids were identified by PCR and sequencing after plasmid DNA were extracted.^18^ Then, eight vectors above were sent to Shanghai Shenggong Company for sequencing and identification.

### 2.3 Generation and passage of rFLU‐P1a and rFLU‐P30a viruses

Eight pHW2000 plasmids containing the NS‐P1a or NS‐P30a segment and the other seven PR8 influenza virus genomic segments were transfected into 293 T cells using Lipofectamine® 3000 transfection kit (Invitrogen) and incubated with Opti‐MEM at 37°C more than 6 h. The DNA mixture was removed, and DMEM medium containing FBS was added. 72 h later, the viral supernatant was collected and inoculated into the allantoic cavity of 9–11 day old chicken embryos. The recombinant influenza virus strains (named rFLU‐P1a and rFLU‐P30a, respectively) were cultured at 34°C for 72 h. Then the allantoic fluid containing virus was collected, designated rFLU‐P1a/P30a passage 1 virus, and was subsequently passaged in SPF chicken embryos at 34°C. The allantoic fluid was harvested 72 h later. Infectious virus titers were determined by erythrocyte agglutination test. The data were represented by mean ± standard deviation (X̅ ± SD) and processed by GraphPad Prism statistical software. The comparison between the two groups was performed by Student's t‐test, and *p* < 0.05 indicated statistically significant difference.

### 2.4 Identification of recombinant influenza virus rFLU‐P1a and rFLU‐P30a

For the rescued viruses, insertion of the P1a or P30a gene were confirmed by reverse transcription ‐polymerase chain reaction (RT‐PCR) using NS‐specific and P1a/P30a specific primers. Total RNA from allantoic fluid above was extracted using the TIANamp Virus RNA Kit (TIANGEN Biotech) according to the manufacturer's protocol and then converted to cDNA by Reverse Transcriptase M‐MLV kit (Takara). The cDNA was then subjected to RT‐PCR using primers that are specific to the target sequence (P1a forward‐5'‐CTCAAAACAAC GACACCGGTATT−3'; P1a reverse−5'‐CTGGTTAAACGGACTAAACAAGGTT‐3'; P30a forward−5'‐ATGCTAGTGCTGTTCAGCGCT‐3'; P30a reverse−5'‐GCGTTTTGGTGGAAAACC G−3'; NS forward−5'‐ ACCTCCGAAGTTGGGGGGGAGCAAAAGCAGGGTG ‐3'; NS reverse−5'‐ TGGGCCGCCGGGTTATTAGTAGAAACAAGGGTGTTTT ‐3', respectively). RT‐PCR was performed under the following reaction conditions: 95°C for 5 min, followed by 30 cycles of 95°C for 10 s, 55°C for 10 s and 72°C for 2 min, and 72°C for 8 min. The presence of inserted sequences in generated vaccine virus was further confirmed by Sanger sequencing.

### 2.5 Observation of the morphology of rFLU‐P1a and rFLU‐P30a under transmission electron microscopy

Take the rescued recombinant influenza viruses rFLU‐P1a and rFLU‐P30a for negative staining and observe them under a transmission electron microscope (HITACHI‐7650). The specific steps are as follows: (1) Drip 5 μL of the sample onto a copper mesh for 5 min, and draw the excess liquid away with filter paper; (2) Drip 5 μL dye solution (2% phosphotungstic acid solution, pH 6.5) onto a copper mesh for 5 min. Draw the excess liquid away with filter paper; (3) Dry for about 30 min and observe under transmission electron microscope H‐7650.

## 3 RESULTS

### 3.1 Construction of pHW2000 vector containing PR8 influenza virus 8 fragment

**Figure 3:**
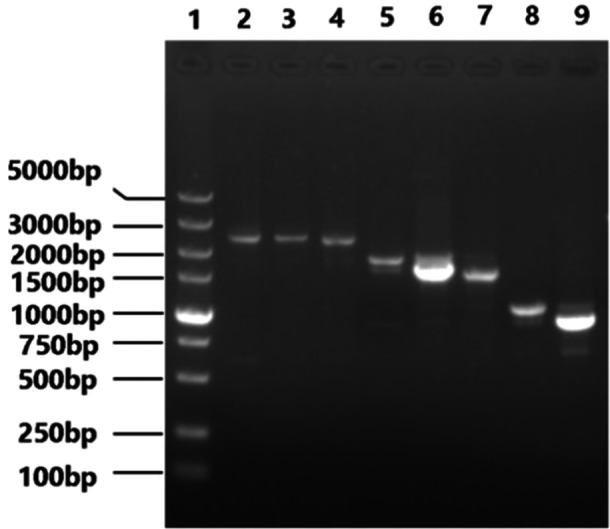
Electrophoretogram of PCR products of eight segments of influenza virus and digested vector. Lane 1: DL 5000 DNA Marker; Lane 2: PB2; Lane 3: PB1; Lane 4: PA; Lane 5: HA; Lane 6: NP; Lane 7: NA; Lane 8: M; Lane 9: NS.

**Figure 4:**
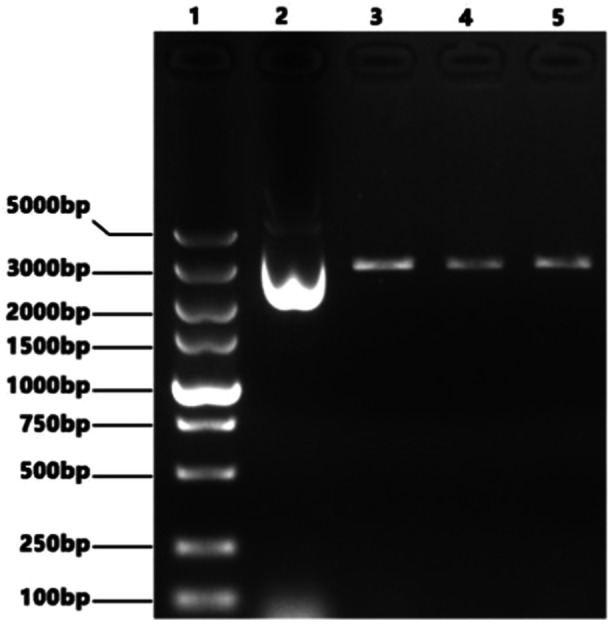
BsmBⅠ digested pHW2000 vector in electrophoresis. Lane 1: DL5000 DNA Marker; Lane 2: pHW2000 plasmid before enzyme digestion; Lane3‐5: pHW2000 plasmid after BsmB Ⅰ enzyme digestion.

Perform RT‐PCR amplification on the eight fragments of PB2(2280 bp), PB1(2274 bp), PA(2151 bp), HA(1701 bp), NP(1497 bp), NA(1365 bp), M(982 bp) and NS(890 bp) of the H1N1 PR8 maternal strain. Restriction enzyme BsmB Ⅰ was used to cleave pHW2000 plasmids. After gel recovery of PCR products and enzyme digestion vectors above, we use 1% agarose gel electrophoresis for nanalysis and identification, as shown in Figures 3 and 4 below.

**Figure 5:**
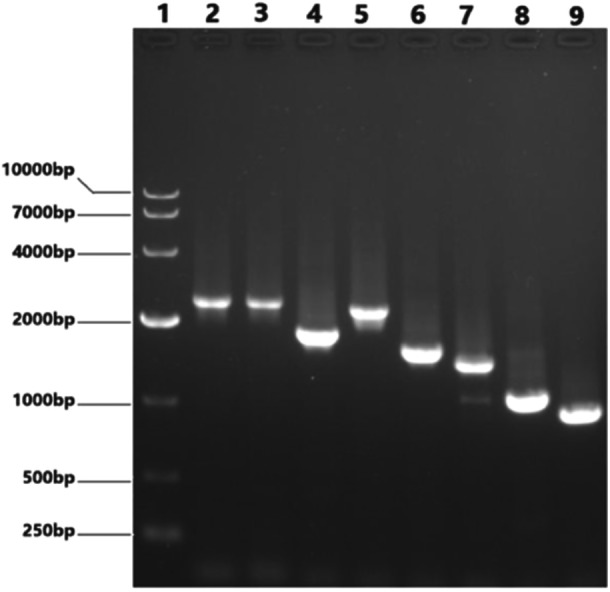
Eight segments of H1N1 PR8 in electrophoresis. Lane 1: DL10000 DNA Marker；Lane 2: PB2；Lane 3: PB1；Lane 4: HA；Lane 5: PA; Lane 6: NP; Lane 7: NA; Lane 8: M; Lane 9: NS.

After ligation by Exnase Ⅱ, the recombinant plasmids were transformed into DH5α competent cells and were identified by PCR after plasmid DNA were extracted. The results were shown in Figure 5 below.

**Figure 6:**
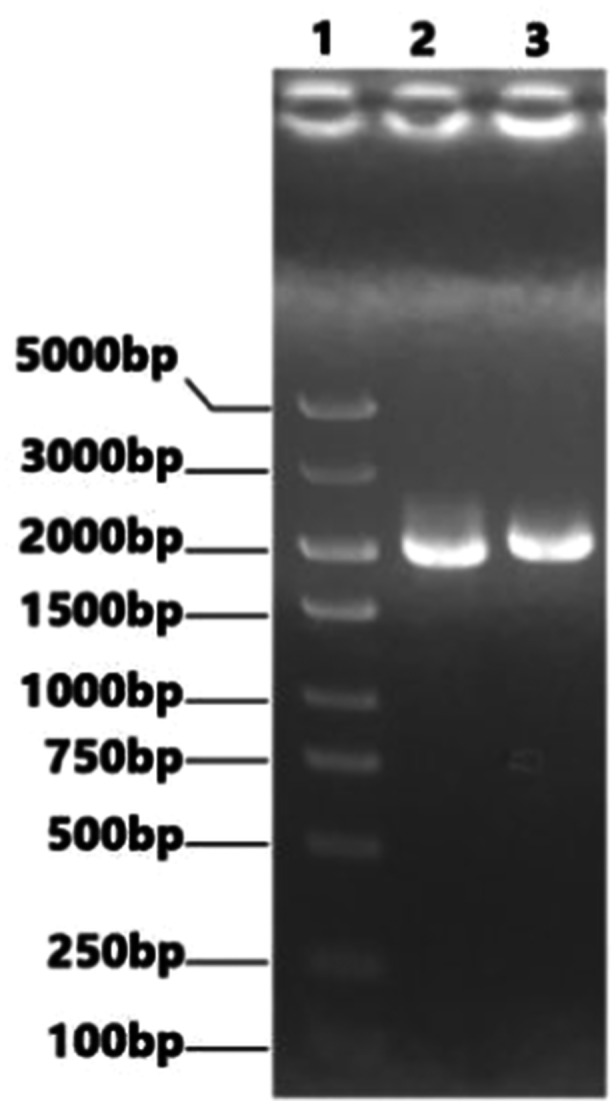
NS‐P1a, NS‐P30a PCR identification in electrophoresis. Lane 1: DL5000 DNA Marker; Lane 2: NS‐P1a band (1992bp); Lane 3: NS‐P30a band (2073 bp).

The sequences of the recombinant NS fragment inserted with P1a, P30a and the modified sequences were designed and sent with pHW2000 plasmid to Bioengineering (Shanghai) Co., Ltd. for synthesis and sequencing analysis. The synthesized recombinant vectors pHW‐NS‐P1a and pHW‐NS‐P30a were identified by PCR with NS primers. The agarose gel electrophoresis diagram is shown in Figure 6 below.

### 3.2 Rescue and identification of recombinant influenza viruses rFLU‐P1a and rFLU‐P30a

**Figure 7:**
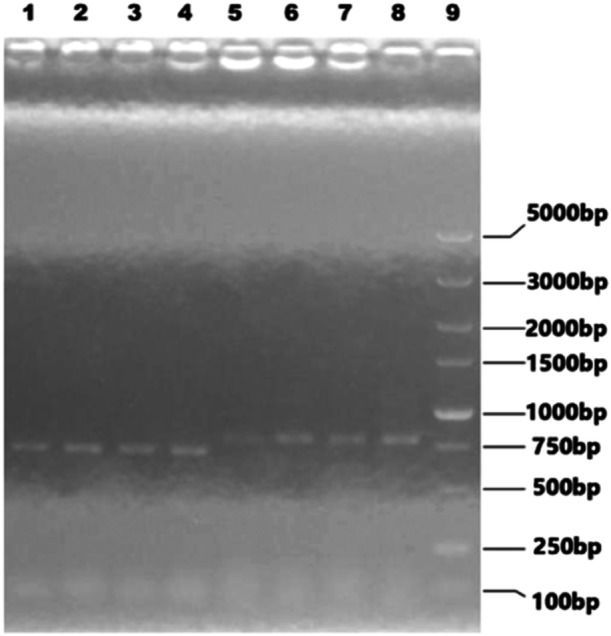
P1a, P30a PCR products in electrophoresis. Lane 1‐4: P1a band (693 bp); Lane 5‐8: P30a band (774 bp); Lane 9: DL5000 DNA Marker.

Selecting recombinant plasmids containing 7 fragments of PR8 virus (PB2, PB1, HA, PA, NP, NA, M) and pHW‐NS‐P1a/pHW‐NS‐P30a as the “7 + 1” combination to co‐transfect HEK293T cells. Then 9‐10 day old SPF chicken embryos were inoculated with transfected harvest solution above for 72 h at 34°C. Collecting the chicken embryo allantoic fluid separately, hemagglutination (HA) titers were determined by erythrocyte agglutination test. The result showed that HA titer of rFLU‐P1a was 1: 128, and the HA titer of rFLU‐P30a was 1: 32. rFLU‐P1a and rFLU‐P30a were identified by RT‐PCR using specific primers of P1a and P30a, respectively. The electrophoresis results were shown in the following Figure 7.

### 3.3 Study on the stability of rFLU‐P1a and rFLU‐P30a in continuous passage in chicken embryos

rFLU‐P1a and rFLU‐P30a recombinant viruses were transmitted to chicken embryos for five successive generations, and the hemagglutination titers of viruses in allantoic fluid were measured and compared. The results were shown in the following Table 1 (**indicates a significant difference compared to the rFU‐P1a group, *p* < 0.01.).

**Table 1:** Hemagglutination titer results of recombinant virus for five generations.

Chicken embryo generation virus species	1	2	3	4	5	x̅ ± SD
rFLU‐P1a	1: 128	1: 64	1: 128	1: 128	1: 128	1: (115.2 ± 28.62)
rFLU‐P30a	1: 32	1: 32	1: 64	1: 32	1: 32	1: (38.40 ± 14.31)**

*Note*: **indicates a significant difference of Hemagglutination titer results of recombinant virus for five generations compared rFLU‐P30a group with the rFLU‐P1a group, *p* < 0.01. The P value is 0.0007. The comparison between the 2 groups was performed by Student's t‐test, and *P* < 0.05 indicated statistically significant difference.

**Figure 8:**
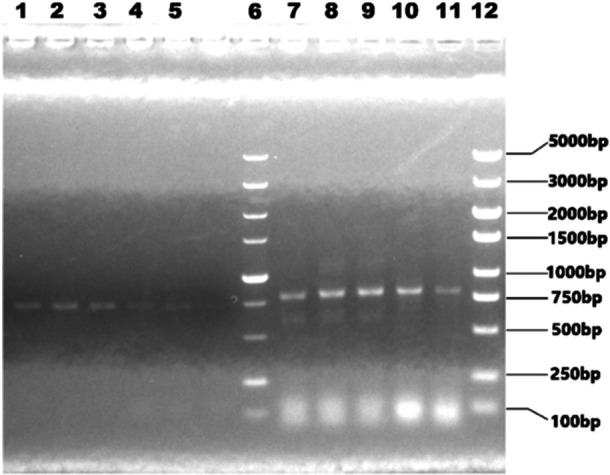
RT‐PCR products identification in electrophoresis. Lane 1‐5: P30a band (774 bp) of the 1st to 5th generation rFLU‐P30a viruses; Lane 6: DL5000 DNA Marker; Lane 7‐11: P1a band (693 bp) of the 1st to 5th generation rFLU‐P1a viruses; Lane 12: DL5000 DNA Marker. RT‐PCR, reverse transcription ‐polymerase chain reaction.

RNA of rFLU‐P1a and rFLU‐P30a were extracted from the chicken embryo allantoic fluid of 5 consecutive generations, and the cDNA which obtained by reverse transcription was identified by PCR using the specific primers of P1a and P30a, respectively. The electrophoresis results were shown in the following Figure 8.

From the above results, it can be seen that after 5 successive generations, each virus generation has a certain hemagglutination titer, and the band of P1a or P30a can be seen in the corresponding positions, which indicating that the genetic stability of the recombinant virus is relatively high.

### 3.4 Observation of the morphology of recombinant influenza viruses rFLU‐P1a and rFLU‐P30a under electron microscopy

**Figure 9:**
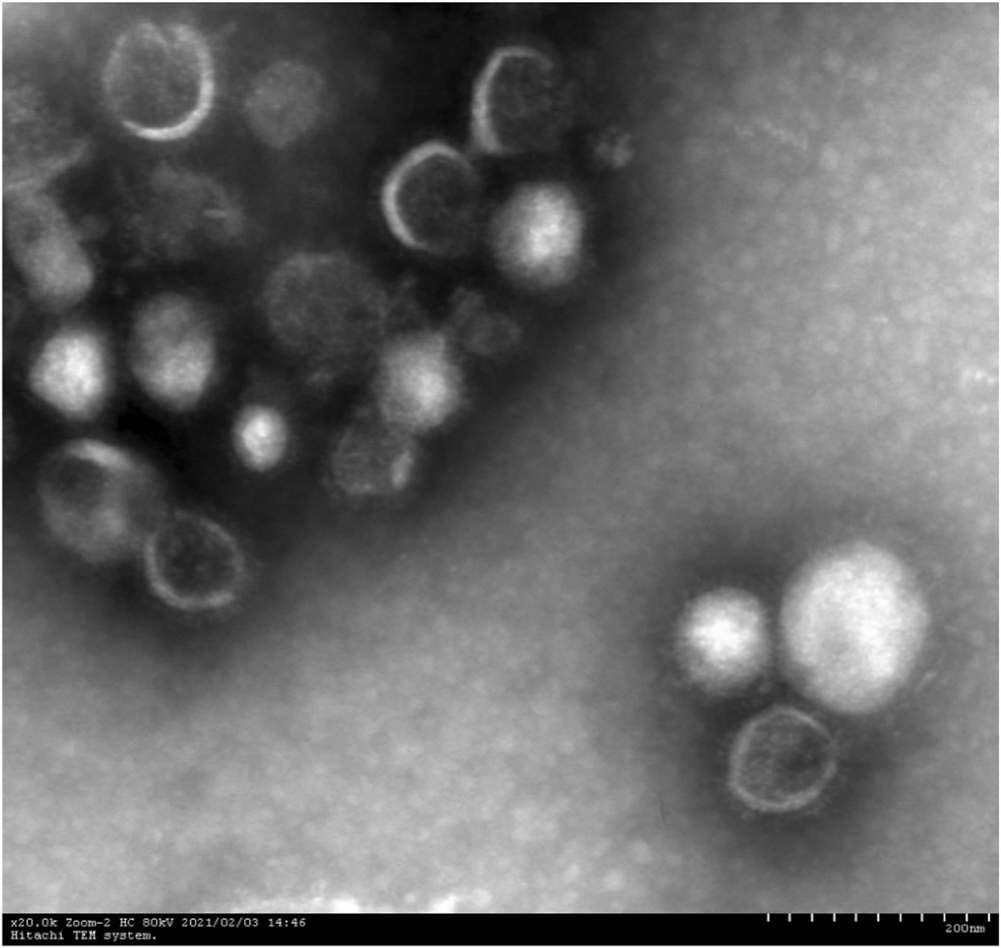
Electron micrograph of rFLU‐P1a virus (×20.0 K).

**Figure 10:**
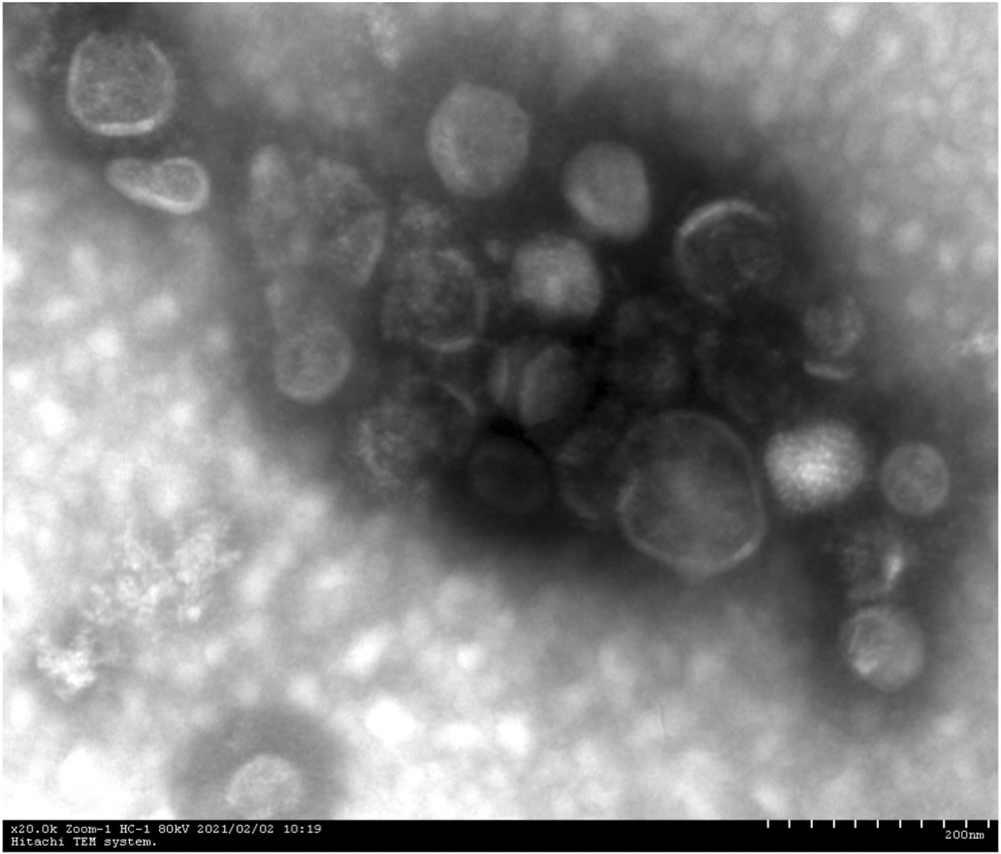
Electron micrograph of rFLU‐P30a virus (×20.0 K).

After negative staining of the rescued recombinant influenza viruses rFLU‐P1a and rFLU‐P30a, they were observed under an electron microscope. The typical and complete morphology of influenza virus was observed under the electron microscope, as shown in the figures below(Figures 9 and 10).

The virus particles appeared as spheres or long strips connected by several particles, revealing a complete viral membrane structure composed of virus lipid bilayer, hemagglutinin, neuraminidase, and matrix proteins.

## 4 DISCUSSION

To date, the *MP* vaccine for human use is still in the research and development stage, and vaccine research hotspots mainly focus on the whole cell antigen, adhesion protein antigen and community acquired respiratory distress syndrome (*CARDS*) toxin of *MP*.^19^ All *MP* vaccines mentioned above are administered by intramuscular injection to elicit the production of primarily serum neutralizing antibodies and systemic T cell responses to fight against *MP* infection.^11^ However, intramuscular vaccines induce poor local immunity in the respiratory tract, which is the primary infection site for *MP*.^20^ According to the data of a meta‐analysis,^21^ the summarized efficacy of MP vaccines against pneumonia regardless of etiologies was 36%, the efficacy of the vaccines against MP‐associated pneumonia was 42%, and inactivated MP vaccines may reduce the total rates of both pneumonia and respiratory infections by approximately 40%. Therefore, redeveloping MP vaccines is necessary, particularly for high‐risk settings as well as in the general population. One solution to achieve more effective prevention of *MP* infection is to enhance the local immunity in the respiratory tract.^22^ A similar study showed immune responses induced by an NS1‐deleted influenza virus vectored intranasal *COVID‐19* vaccine (*dNS1‐RBD*) which provides broad‐spectrum protection against *SARS‐CoV‐2* variants in hamsters.^23^ In this study, the major antigen genes P1a and P30a of *MP* adhesion factor P1 and P30 were inserted into the nonstructural protein (NS) gene of PR8 to construct the recombinant vectors NS‐P1a or NS‐P30a. The recombinant plasmids were cotransfected with the rest 7 fragments of *PR8* into *HEK293T* cells. After inoculating chicken embryos, the recombinant influenza viruses rFLU‐P1a and rFLU‐P30a were rescued. RT‐PCR identification of the recombinant virus showed that P1a (693 bp), P30a (774 bp), NS‐P1a (1992bp) and NS‐P30a (2073 bp) bands were found, and the sequencing results were correct.

It is widely known that increasing the number of passages of recombinant influenza virus in chicken embryos increases the possibility of antigenicity drift.^24^ Therefore, in this experiment, two recombinant virus strains rFLU‐P1a and rFLU‐P30a were inoculated on chicken embryos for five consecutive passages. The hemagglutination titers of rFLU‐P1a and rFLU‐P30a were basically stable at 1:128 and 1:32, and RT‐PCR identification of the virus solution harvested from five passages revealed P1a and P30a bands, which confirming their high genetic stability. Continuous passages showed no death of chicken embryos after virus inoculation, which indicates good safety of the recombinant virus. Thus, the rFLU‐P1a and rFLU‐P30a can be used for subsequent large‐scale cultivation and posttreatment purification research. Further studies should be conducted to understand the immunogenicity and protection mechanism especially in intranasal immunization of rFLU‐P1a and rFLU‐P30a for *MP* infection or transmission in humans.

There are two main limitations in this study. First, Many previous studies utilized a NS1‐deleted (*dNS1*) influenza viral vector to engineer an live‐attenuated vectored vaccine,^23^, ^25^ whereas in this study we used the whole NS fragment without deleting NS1. This may limit the length of the inserted target gene and may affect the success rate of recombination and the effectiveness of the recombinant virus. Second, although our study creatively explored the possibility of using influenza virus strain as a vector to develop *MP* genetically engineered live‐attenuated vaccines and we have successfully constructed and rescued recombinant influenza viruses containing dominant antigen fragments of MP P1 and P30, it is important to note that the immunogenicity and duration of protection needs to be confirmed in more animal models in the future.

## AUTHOR CONTRIBUTIONS

**Liang Yu:** Data curation; formal analysis; funding acquisition; methodology; project administration; resources; software; supervision; validation; visualization; writing—original draft. **Wang Yongbo:** Methodology; visualization; writing—review and editing. **Yang Shengjun:** Investigation; software; visualization; writing—review and editing. **Tan Jia:** Formal analysis; validation; writing—review and editing. **Xu Ya:** Formal analysis; methodology; software; visualization; writing—review and editing. **Liao Guoyang:** Conceptualization; funding acquisition; methodology; project administration; resources; supervision; validation; writing—review and editing. **Ma Linna**: Data curation; formal analysis; investigation; software; supervision; validation; visualization; writing—review and editing.

## CONFLICT OF INTEREST STATEMENT

The authors declare no conflict of interest.
